# A mini-review of mathematical methods in sprint performance

**DOI:** 10.3389/fspor.2025.1696505

**Published:** 2025-11-10

**Authors:** Yuzhou Fan

**Affiliations:** 1 School of Physical Education and Sports Science, Fujian Normal University, Fuzhou, China; 2 Shenzhen Campus, Jinan University, Shenzhen, China

**Keywords:** mathematics, sport science, sprint performance, mathematical modeling, biomechanics, machine learning

## Abstract

Mathematics has established itself as a core analytical tool in sprint performance research within sports science, offering quantitative insights that inform coaching strategies, training methodologies, and athlete development. This mini-review examines eight highly-cited publications by Peter Wey and and colleagues, whose work has significantly advanced understanding of sprint biomechanics through the integration of mathematical and biomechanical modeling approaches. This review analyzes diverse methodological applications, ranging from regression models for predicting athletic potential to differential equations for kinetic and kinematic analysis of sprint mechanics. Critical evaluation of these seminal studies demonstrates how mathematical approaches provide objective frameworks for performance analysis, enhance predictive capabilities, and offer mechanistic insight into sprint performance determinants. Then findings underscore the fundamental role of mathematical methods in advancing spring performance research and highlight opportunities for further methodological development in sports science applications.

## 1 Introduction

Sport science is undergoing a fundamental transformation driven by the convergence of biomechanics, physiology, and advanced analytics, with mathematics serving as a linking mechanism across disciplines—offering a rigorous quantitative framework for analyzing, predicting, and optimizing athletic performance. By integrating mathematical models and analytical techniques, researchers can identify underlying patterns, assess physiological data, and refine training methodologies. These models are instrumental in examining movement mechanics, force generation, and energy expenditure in various sports, ultimately providing a scientific basis for performance optimization (1–4). As disciplines become increasingly mathematical in nature, their scientific value and credibility tend to grow correspondingly (5). In the contemporary era characterized by advancements in artificial intelligence (AI) and machine learning (ML), mathematics has assumed an unprecedented significance in sport science (6). This is particularly evident in areas such as injury prediction accuracy, performance analysis precision, training program customization, and the overall improvement in athletic performance (7).

Mathematics encompasses several core branches, including number theory, algebra, geometry, analysis, and set theory, etc. (8). Within this broad field, sport statistics represents a specialized application of mathematical principles. In sport science, statistical analysis is essential for evaluating athletic performance, informing training strategies, optimizing tactics, guiding player recruitment, and supporting effective sports management (9). Given its significance, sport statistics is a fundamental component of higher education curricula, serving as a required course for students pursuing degrees in physical education and sports training (10).

Statistics indeed enables us to identify patterns and trends within data by systematically analyzing and interpreting it. However, its reliability is often overestimated by many scientists (11). Moreover, the binary interpretation of *p*-values (significant vs. not significant) does not account for the magnitude of effect size or the true significance of results (12). The “*p*-value fallacy” highlights how *p*-values can be manipulated or misinterpreted, particularly in evidence-based medicine (13, 14). This is not necessarily an act of forgery but rather a methodological choice, which justifies its widespread application. Therefore, *p*-values should not be relied upon in isolation as definitive evidence for supporting a model or hypothesis in sport science.

Mathematical approaches in sports science encompass a diverse range of quantitative methods designed to analyze, model, and optimize human athletic performance through systematic data analysis and computational techniques. Biomechanical modeling involves the application of physics and engineering principles to understand human movement patterns, joint forces, and energy expenditure during athletic activities, providing insights into technique optimization and injury prevention mechanisms (15). Statistical analysis serves as the foundation for performance evaluation, utilizing descriptive statistics, correlation analysis, and regression modeling to identify relationships between training variables and performance outcomes, enabling evidence-based decision-making in athletic preparation (16). Machine learning algorithms, including supervised learning methods such as decision trees, random forests, and neural networks, analyze complex datasets to predict performance trends, classify movement patterns, and personalize training interventions based on individual athlete characteristics (17). Time-series analysis examines performance data collected over extended periods to identify trends, seasonal variations, and training adaptations, helping coaches understand long-term athletic development and optimize periodization strategies (18). Optimization techniques employ mathematical algorithms to determine optimal training loads, recovery periods, and competition strategies by maximizing performance outcomes while minimizing injury risk through data-driven modeling approaches that integrate physiological constraints with performance objectives (19). Signal processing methods filter and analyze physiological data from wearable sensors, heart rate monitors, and GPS devices to extract meaningful performance indicators and eliminate noise from raw measurement data (20, 21). These mathematical frameworks collectively provide sports scientists with quantitative tools to transform raw performance data into actionable insights, though their methodological diversity and application-specific nature often preclude standardized implementation across different sporting contexts.

Sprint performance has long fascinated sport scientists, who strive to unravel the complex interplay between biomechanics, physiology, and morphology that underpins world-class speed (22–24). In recent decades, mathematical methods have emerged as foundational tools in deciphering these elements—not merely as adjuncts to statistics but as powerful engines for modeling, prediction, and theoretical innovation (25). Mathematical applications in sprint performance research encompass diverse methodologies and heterogeneous analytical approaches, precluding quantitative synthesis through meta-analysis (26, 27). Despite substantial research activity, no comprehensive review has synthesized how mathematics contributes to understanding sprint biomechanics and performance optimization (28, 29). This knowledge gap is particularly significant given the increasing integration of advanced mathematical tools in sports science practice (30, 31). Due to the methodological diversity across mathematical modeling studies (32, 33) and the limited number of directly comparable investigations (34, 35), a pilot study of narrative mini review focusing on conceptual synthesis rather than statistical pooling will provide researchers, coaches and practitioners with accessible guidance on applying mathematical insights to sprint performance enhancement (36, 37).

Among prominent researchers in sprint biomechanics, Peter Weyand's contributions are particularly noteworthy for establishing foundational mathematical frameworks, including force-velocity relationships and metabolic power models that continue to influence contemporary research. The wide application and high citation impact of Weyand's mathematical approaches provide an ideal foundation for examining the evolution and practical utility of quantitative methods in sprint performance analysis. Drawing from eight highly cited papers (co-)authored by Peter Weyand and colleagues, this mini review explores how diverse mathematical strategies—from force-time curve analysis to computational simulations—propel our understanding of sprinting to new levels of accuracy and reliability, revealing both the rigor of the field and a promising roadmap for future research in human performance.

## 2 Methods

This mini-review employed a purposive sampling approach to examine mathematical applications in sprint performance research through the lens of influential biomechanical investigations. A three-stage selection protocol was implemented to ensure methodological rigor and thematic coherence.

### 2.1 Selection criteria

#### 2.1.1 Primary author criterion

Studies were required to include Peter Weyand as an author (first, corresponding, or co-author), based on his established expertise in locomotion biomechanics and physiology. Weyand's scholarly impact (>6,000 citations, h-index of 32) and specialized focus on human and animal locomotion provided a coherent theoretical framework for examining mathematical approaches in sprint research.

#### 2.1.2 Content inclusion criteria

Papers must explicitly address one or more core biomechanical parameters of human sprinting: (a) ground reaction forces and contact mechanics, (b) metabolic energy expenditure and efficiency, and (c) velocity-related kinematic variables. Studies examining these parameters in relation to sprint performance optimization were prioritized.

#### 2.1.3 Methodological requirements

Selected studies must demonstrate explicit application of mathematical or computational methods, including but not limited to: biomechanical modeling, statistical regression analysis, kinematic simulations, force-velocity profiling, or energy cost calculations.

### 2.2 Search and selection process

From Weyand's complete publication corpus, identified through Google Scholar (*n* > 100) and Web of Science All Databases (Au = Weyand P*; *n* > 50), studies were initially screened by title and abstract (*n* = 20) for relevance to sprint biomechanics. Full-text review was then conducted to assess mathematical methodology application and sprint-specific content.

### 2.3 Final sample

Eight studies meeting all inclusion criteria were purposively selected for comprehensive analysis, representing a focused examination of mathematical approaches across key biomechanical domains in sprint performance research. Table 1 provides a detailed overview of these selected studies.

**Table 1:** Basic information of eight selected studies on sprint performance.

No.	Papers selected	Citations^a^/^b^	Paper title
1	(38)	821/1,395	Faster top running speeds are achieved with greater ground forces not more rapid leg movements
2	(45)	244/423	The biological limits to running speed are imposed from the ground up
3	(39)	135/227	The fastest runner on artiﬁcial legs: different limbs, similar function?
4	(40)	135/222	Are running speeds maximized with simple-spring stance mechanics?
5	(43)	97/160	High-speed running performance: a new approach to assessment and prediction
6	(41)	91/146	A general relationship links gait mechanics and running ground reaction forces
7	(44)	82/144	Running performance has a structural basis
8	(42)	77/117	High-speed running performance is largely unaffected by hypoxic reductions in aerobic power

a The citation number is retrieved on October 1, 2025 from Web of Science All Databases.

b The citation number is retrieved on August 27, 2025 from (https://scholar.google.com/scholar?hl=en&as_sdt=0%2C5&q=peter+weyand).

## 3 Results

In sprint research by Weyand and colleagues, mathematics transcends descriptive statistics, enabling modeling, prediction, optimization, and engineering design. For example, force-time curve analyses have anchored biomechanical inquiry, allowing both students and scientists to directly engage with calculus, systems modeling, and real kinetic data (38). Comparative computational approaches—such as examining prosthetic vs. biological limb mechanics—bridge engineering, physics, human biology, and even ethics (39). See Table 2 for how these eight highly cited studies exemplify this interdisciplinary, mathematically intensive approach to sport science.

**Table 2:** Mathematical categorization of Weyand et al. research.

Mathematical area	Key techniques & models	Primary applications in the research
Statistical modeling & analysis	Linear Regression, ANOVA, *R*^2^, RMSE	Testing hypotheses and analyzing relationships between ground forces, stride parameters, and speed (38). Validating spring-mass model predictions against measured force data (40). Comparing performance metrics between prosthetic and biological limbs (39).
Dynamical systems & differential equations	Spring-Mass Models, Two-Mass Models, Motion Equations	Developing a two-mass mechanical model to predict vertical ground reaction forces (GRFs) based on body mass and kinematics (41). Critiquing the predictive capacity of single and multi-mass models for GRF waveforms (29).
Exponential decay models	Functions of the form $\mathrm{Spd}=Spd_{aer}+(Spd_{an}-Spd_{aer})\cdot e^{-kt}$	Modeling the decline in sprint running speed over durations from 3 to 240 s (43). Quantifying the effects of hypoxia on anaerobic energy release and sprint performance (42).
Algebraic & geometric relationships	Dimensional Analysis, Scaling Laws (e.g., $M_{b}=F_{g}\times H^{2}\times D$)	Deriving a fundamental scaling law that explains how stature and body mass determine mass-specific force production capacity in runners (44).
Kinetic & kinematic equations	Force-Velocity Relationships, Critical Power Framework, Stance-Averaged Force Equations	Applying stance-averaged force calculations to determine the mechanical limits to running speed (45).
Model validation metrics	Coefficient of Determination (*R*^2^), Root Mean Square Error (RMSE)	Quantifying the accuracy of the two-mass model's force predictions against experimental data (e.g., *R*^2^ = 0.95 in (41)

## 4 Discussion

The collective work of Weyand and colleagues presents a compelling case for the indispensable role of mathematical modeling in advancing our understanding of human locomotion. The research program moves beyond qualitative description, employing a suite of quantitative techniques to dissect the fundamental principles governing running performance. This integrative approach allows for the formulation of general, predictive theories rather than merely documenting observed phenomena.

The foundational layer of this work is built upon **statistical modeling**, which provides the critical link between theory and experiment. The consistent use of linear regression and ANOVA across studies (38, 39) rigorously establishes the relationships between key biomechanical variables, such as contact time and ground reaction force. More importantly, metrics like the coefficient of determination (*R*^2^) and root mean square error (RMSE) (40, 41) serve as objective, quantitative benchmarks for model validity. This transforms theoretical models from conceptual frameworks into tools with tested predictive power.

At the core of the mechanistic explanations are **dynamical systems** formulated through differential equations. The progression from simple spring-mass models to more sophisticated two-mass representations (29, 41) exemplifies the iterative process of scientific modeling. Each model embodies a specific hypothesis about how the body generates force, and its failure or success in predicting experimental GRF waveforms (as quantified by *R*^2^) directly informs physiological understanding. These models successfully isolate the primary mechanical determinants of performance—body mass, leg acceleration, and contact time—and describe their interactions through mathematical laws.

For modeling performance over time, **exponential decrements functions** have proven highly effective. The repeated successful application of the form $\mathrm{Spd}=Spd_{aer}+(Spd_{an}-Spd_{aer})\cdot e^{-kt}$ to model the decline in speed and anaerobic energy (42, 43) suggests that a fundamental, first-order process governs high-intensity energy utilization. The decay constant *k* provides a quantitative measure of fatigue resistance, offering a powerful metric to assess the impact of interventions like hypoxia or to compare different athlete populations.

Perhaps the most elegant finding is the derivation of a simple **algebraic scaling law** (44). The relationship $M_{b}=F_{g}\times H^{2}\times D$ demonstrates that complex performance outcomes can sometimes be distilled into a concise mathematical principle. This equation effectively reconciles how runners of vastly different sizes can achieve similar performance levels by revealing the underlying structural proportionality between stature, mass, and force production.

Finally, the work leverages **kinetic equations** to establish the limits of performance. By applying Newtonian mechanics to stance-phase dynamics, Weyand et al. (45) translated a biomechanical observation—that faster speeds are achieved with greater ground forces, not more rapid leg cycling—into a quantifiable mechanical limit.

While the models presented are powerful, they inevitably involve simplification. The two-mass model, for instance, simplifies the complex, multi-segmented human body into two-point masses (41). Future research could explore more complex musculoskeletal models to capture finer details of the force-time waveform. Furthermore, the parameters within the exponential decay models (e.g., *k*) are phenomenological; their precise physiological correlates—whether related to metabolite accumulation, neuromuscular fatigue, or other factors—remain a rich area for investigation.

In addition, AI has transformed sports science from basic performance analytics to sophisticated, data-driven decision-making systems that revolutionize athletic training and performance optimization (Mateus et al., 2024). AI applications in sprint research include predictive modeling, real-time analytics, computer vision tracking systems, and hybrid models like Convolutional Neural Network—Long Short-Term Memory (CNN-LSTM) for analyzing movement patterns and predicting sprint success [(46); Mateus et al., 2024; (47, 48)]. These AI tools, including ML models such as random forests and gradient boosting algorithms, enable precise athlete profiling, automated movement analysis, and personalized training programs that optimize performance while reducing injury risk (17, 48, 49). However, the diverse range of AI methods creates challenges for standardization and evaluation, while the complexity of human physiological responses makes traditional meta-analytical approaches difficult, requiring collaboration between sports scientists, data scientists, and AI engineers [(50); Mateus et al., 2024]. Future developments in AI-driven sprint analysis will enhance personalized coaching, injury prevention, and real-time feedback, but require rigorous validation studies and standardized frameworks to fully realize their potential in evidence-based athletic optimization (48, 51).

This pilot review adopted a focused methodological framework that prioritized conceptual clarity and accessibility over comprehensive design diversity. While mathematical approaches in sprint performance served as illustrative examples to demonstrate foundational principles, the scope was intentionally constrained to facilitate understanding of mathematical literacy applications. By employing AI and ML technologies, future systematic reviews should incorporate a broader methodological spectrum, including longitudinal cohort studies, randomized controlled trials, cross-sectional analyses, case-control studies, and mixed-methods approaches. Additionally, expanding beyond sprint performance to encompass diverse athletic disciplines would strengthen the generalizability of mathematical applications in sports science. Such methodological expansion would provide a more comprehensive evidence base and enhance the robustness of findings across varied research contexts.

In conclusion, this mini-review demonstrates that mathematical approaches are fundamental to advancing sprint performance research and applied practice. Through systematic analysis of eight highly-cited publications by Weyand and colleagues, it has been illustrated how diverse mathematical methods, including regression analysis, biomechanical modeling, force-velocity profiling, and kinematic analysis, etc., provide objective, quantifiable frameworks for understanding the determinants of sprint performance. The precision and predictive capacity of these mathematical models have direct implications for elite sports, informing evidence-based training interventions, enabling individualized performance assessments, and guiding the development of performance enhancement strategies. Future research should build upon these foundational approaches by integrating emerging computational methodologies and expanding mathematical modeling to address evolving questions in sprint biomechanics, ultimately bridging the gap between theoretical understanding and practical performance optimization in elite athletics. As the field advances, researchers and practitioners should proactively prepare for the integration of AI technologies, which will play an increasingly indispensable role in sprint performance optimization specifically and in transforming the landscape of sports science more broadly.

## References

[1] Blickhan R. The spring-mass model for running and hopping. J Biomech. (1989) 22:1217–27. 10.1016/0021-9290(89)90224-8 2625422

[2] Vincenzo C Sandra S. Scaling laws and forecasting in athletic world records. J Sports Sci. (2001) 19:477–84. 10.1080/026404101750238935 11461051

[3] Ward-Smith AJ Radford PF. A mathematical analysis of the 4× 100 m relay. J Sports Sci. (2002) 20:369–81. 10.1080/026404102317366627 12043826

[4] Orange ST Hritz A Pearson L Jeffries O Jones TW Steele J. Comparison of the effects of velocity-based vs. Traditional resistance training methods on adaptations in strength, power, and sprint speed: a systematic review, meta-analysis, and quality of evidence appraisal. J Sports Sci. (2022) 40:1220–34. 10.1080/02640414.2022.2059320 35380511

[5] Resnik MD. Mathematics as a Science of Patterns. New York: Oxford University Press (1997).

[6] Krstić D Vučković T Dakić D Ristić S Stefanović D. The application and impact of artificial intelligence on sports performance improvement: a systematic literature review. 2023 4th International Conference on Communications, Information, Electronic and Energy Systems (CIEES); Plovdiv, Bulgaria: IEEE (2023). p. 1–8. 10.1109/CIEES58940.2023.10378750

[7] Reis FJJ Alaiti RK Vallio CS Hespanhol L. Artificial intelligence and machine learning approaches in sports: concepts, applications, challenges, and future perspectives. Braz J Phys Ther. (2024) 28:101083. 10.1016/j.bjpt.2024.101083 38838418 PMC11215955

[8] Neukirch J. Algebraic Number Theory. Verlag Berlin Heidelberg New York: Springer Science & Business Media (2013).

[9] Šiljak V ana Mijatović S Gavrilović D. Problems of statistics application in sports science. Sci Int J. (2024) 3:21–5. 10.35120/

[10] Weir JP Vincent WJ. Statistics in Kinesiology. Champaign: Human Kinetics (2021).

[11] Nuzzo R. Scientific method: statistical errors. Nature. (2014) 506:150–2. 10.1038/506150a 24522584

[12] Wasserstein RL Lazar NA. The ASA statement on p -values: context, process, and purpose. Am Stat. (2016) 70:129–33. 10.1080/00031305.2016.1154108

[13] Goodman SN. Toward evidence-based medical statistics. 1: the *p* value fallacy. Ann Intern Med. (1999) 130:995–1004. 10.7326/0003-4819-130-12-199906150-00008 10383371

[14] Goel H Raheja D Nadar SK. Evidence-based medicine or statistically manipulated medicine? Are we slaves to the P-value? Postgrad Med J. (2024) 100:451–60. 10.1093/postmj/qgae012 38330498

[15] Winter DA. Biomechanics and Motor Control of Human Movement. 4th Ed Hoboken, NJ: John Wiley & Sons (2009).

[16] Hopkins WG Marshall SW Batterham AM Hanin J. Progressive statistics for studies in sports medicine and exercise science. Med. Sci. Sports Exerc. (2009) 41:3–12. 10.1249/MSS.0b013e31818cb278 19092709

[17] Claudino JG Capanema DDO De Souza TV Serrão JC Machado Pereira AC Nassis GP. Current approaches to the use of artificial intelligence for injury risk assessment and performance prediction in team sports: a systematic review. Sports Med Open. (2019) 5:28. 10.1186/s40798-019-0202-3 31270636 PMC6609928

[18] Impellizzeri FM Marcora SM Coutts AJ. Internal and external training load: 15 years on. Int J Sports Physiol. Perform. (2019) 14:270–3. 10.1123/ijspp.2018-0935 30614348

[19] Malone JJ Lovell R Varley MC Coutts AJ. Unpacking the black box: applications and considerations for using GPS devices in sport. Int J Sports Physiol Perform. (2017) 12:S2-18–26. 10.1123/ijspp.2016-0236 27736244

[20] Seshadri DR Li RT Voos JE Rowbottom JR Alfes CM Zorman CA Wearable sensors for monitoring the physiological and biochemical profile of the athlete. NPJ Digit Med. (2019) 2:72. 10.1038/s41746-019-0150-9 31341957 PMC6646404

[21] Schumann M Doherty C. Bridging gaps in wearable technology for exercise and health professionals: a brief review. Int. J. Sports Med. (2024) 45:949–57. 10.1055/a-2376-6332 39079705

[22] Haugen T Seiler S Sandbakk Ø Tønnessen E. The training and development of elite sprint performance: an integration of scientific and best practice literature. Sports Med Open. (2019) 5:44. 10.1186/s40798-019-0221-0 31754845 PMC6872694

[23] Haugen T McGhie D Ettema G. Sprint running: from fundamental mechanics to practice—a review. Eur J Appl Physiol. (2019) 119:1273–87. 10.1007/s00421-019-04139-0 30963240

[24] Hamad MJ Alcaraz PE De Villarreal ES. Effects of combined uphill–downhill sprinting versus resisted sprinting methods on sprint performance: a systematic review and meta-analysis. Sports Med. (2024) 54:185–202. 10.1007/s40279-023-01916-y 37658966

[25] Lin YC Pandy MG. Predictive simulations of human sprinting: effects of muscle-tendon properties on sprint performance. Med Sci Sports Exerc. (2022) 54:1961–72. 10.1249/mss.0000000000002978 35736543

[26] Morin J-B Gimenez P Edouard P Arnal P Jiménez-Reyes P Samozino P Sprint acceleration mechanics: the major role of hamstrings in horizontal force production. Front Physiol. (2015) 6:404. 10.3389/fphys.2015.00404 26733889 PMC4689850

[27] Rabita G Dorel S Slawinski J Sàez-de-Villarreal E Couturier A Samozino P Sprint mechanics in world-class athletes: a new insight into the limits of human locomotion. Scand J Med Sci Sports. (2015) 25:583–94. 10.1111/sms.12389 25640466

[28] Bezodis IN Kerwin DG Salo AIT. Lower-limb mechanics during the support phase of maximum-velocity sprint running. Med Sci Sports Exerc. (2008) 40:707–15. 10.1249/MSS.0b013e318162d162 18317373

[29] Clark KP Ryan LJ Weyand PG. Foot speed, foot-strike and footwear: linking gait mechanics and running ground reaction forces. J Exp Biol. (2014) 217:2037–40. 10.1242/jeb.099523 24737756

[30] Haugen T Buchheit M. Sprint running performance monitoring: methodological and practical considerations. Sports Med. (2016) 46:641–56. 10.1007/s40279-015-0446-0 26660758

[31] Samozino P Rabita G Dorel S Slawinski J Peyrot N Saez De Villarreal E A simple method for measuring power, force, velocity properties, and mechanical effectiveness in sprint running. Scand J Med Sci Sports. (2016) 26:648–58. 10.1111/sms.12490 25996964

[32] Cross MR Brughelli M Samozino P Morin J-B. Methods of power-force-velocity profiling during sprint running: a narrative review. Sports Med. (2017) 47:1255–69. 10.1007/s40279-016-0653-3 27896682

[33] Monte A Baltzopoulos V Maganaris CN Zamparo P. Gastrocnemius medialis and vastus lateralis *in vivo* muscle-tendon behavior during running at increasing speeds. Scand J Med Sci Sports. (2020) 30:1163–76. 10.1111/sms.13662 32227378

[34] Nagahara R Naito H Miyashiro K Morin J-B Zushi K. Traditional and ankle-specific vertical jumps as strength-power indicators for maximal sprint acceleration. J Sports Med Phys Fit. (2014) 54:691–9. Web of Science ID: WOS:000347861100002 24739258

[35] Colyer SL Nagahara R Salo AIT. Kinetic demands of sprinting shift across the acceleration phase: novel analysis of entire force waveforms. Scand J Med Sci Sports. (2018) 28:1784–92. 10.1111/sms.13093 29630747

[36] Girard O Mendez-Villanueva A Bishop D. Repeated-sprint ability—part I: factors contributing to fatigue. Sports Med. (2011) 41:673–94. 10.2165/11590550-000000000-00000 21780851

[37] Buchheit M Laursen PB. High-intensity interval training, solutions to the programming puzzle: part I: cardiopulmonary emphasis. Sports Med. (2013) 43:313–38. 10.1007/s40279-013-0029-x 23539308

[38] Weyand PG Sternlight DB Bellizzi MJ Wright S. Faster top running speeds are achieved with greater ground forces not more rapid leg movements. J Appl Physiol. (2000) 89:1991–9. 10.1152/jappl.2000.89.5.1991 11053354

[39] Weyand PG Bundle MW McGowan CP Grabowski A Brown MB Kram R The fastest runner on artificial legs: different limbs, similar function? J Appl Physiol. (2009) 107:903–11. 10.1152/japplphysiol.00174.2009 19541739

[40] Clark KP Weyand PG. Are running speeds maximized with simple-spring stance mechanics? J Appl Physiol. (2014) 117:604–15. 10.1152/japplphysiol.00174.2014 25080925

[41] Clark KP Ryan LJ Weyand PG. A general relationship links gait mechanics and running ground reaction forces. J Exp Biol. (2017) 220:247–58. 10.1242/jeb.138057 27811299

[42] Weyand PG Lee CS Martinez-Ruiz R Bundle MW Bellizzi MJ Wright S. High-speed running performance is largely unaffected by hypoxic reductions in aerobic power. J Appl Physiol. (1999) 86:2059–64. 10.1152/jappl.1999.86.6.2059 10368374

[43] Bundle MW Hoyt RW Weyand PG. High-speed running performance: a new approach to assessment and prediction. J Appl Physiol. (2003) 95:1955–62. 10.1152/japplphysiol.00921.2002 14555668

[44] Weyand PG Davis JA. Running performance has a structural basis. J Exp Biol. (2005) 208:2625–31. 10.1242/jeb.01609 16000532

[45] Weyand PG Sandell RF Prime DNL Bundle MW. The biological limits to running speed are imposed from the ground up. J Appl Physiol. (2010) 108:950–61. 10.1152/japplphysiol.00947.2009 20093666

[46] Gurchiek RD Don HSRA Watagoda LCP McGinnis RS van Werkhoven H Needle AR Sprint assessment using machine learning and a wearable accelerometer. J Appl Physio. (2019) 35:164–9. 10.1123/jab.2018-0107 30676153

[47] Wang J Qin Z Wei Z. Power and velocity performance of swing movement in the adolescent male volleyball players—age and positional difference. BMC Sports Sci Med Rehabil. (2024) 16:111. 10.1186/s13102-024-00898-2 38755687 PMC11097490

[48] Souaifi M Dhahbi W Jebabli N Ceylan Hİ Boujabli M Muntean RI Artificial intelligence in sports biomechanics: a scoping review on wearable technology, motion analysis, and injury prevention. Bioengineering. (2025) 12:887. 10.3390/bioengineering12080887 40868401 PMC12383302

[49] Rossi A Pappalardo L Cintia P Iaia FM Fernàndez J Medina D. Effective injury forecasting in soccer with GPS training data and machine learning. PLoS One. (2018) 13:e0201264. 10.1371/journal.pone.0201264 30044858 PMC6059460

[50] Van Eetvelde H Mendonça LD Ley C Seil R Tischer T. Machine learning methods in sport injury prediction and prevention: a systematic review. J Exp Orthop. (2021) 8:27. 10.1186/s40634-021-00346-x 33855647 PMC8046881

[51] Burenbatu B. Comparative analysis of biomechanical patterns in sprinting: a machine learning approach to optimize running performance in track athletes. Mol Cell Biomech. (2024) 21:321–321. 10.62617/mcb.v21i1.321

